# Peridinin from the Marine Symbiotic Dinoflagellate, *Symbiodinium* sp., Regulates Eosinophilia in Mice

**DOI:** 10.3390/md12041773

**Published:** 2014-03-27

**Authors:** Ken-ichi Onodera, Yuko Konishi, Takahiro Taguchi, Sumio Kiyoto, Akira Tominaga

**Affiliations:** 1 Oceanography Section, Science Research Center, Kochi University, Okoh, Nankoku, Kochi 783-8505, Japan; E-Mail: jm-sumio.kiyoto@kochi-u.ac.jp; 2 Medical Research Center, Kochi University, Okoh, Nankoku, Kochi 783-8505, Japan; E-Mail: jm-konishiy@kochi-u.ac.jp; 3 Laboratory of Human Health and Medical Science, Graduate School of Kuroshio Science, Kochi University, Okoh, Nankoku, Kochi 783-8505, Japan; E-Mails: ttaguchi@kochi-u.ac.jp (T.T.); tominaga@kochi-u.ac.jp (A.T.)

**Keywords:** peridinin, fucoxanthin, delayed-type hypersensitivity, eosinophils, eotaxin, *Symbiodinium*, dinoflagellate

## Abstract

Peridinin and fucoxanthin, which are natural carotenoids isolated from a symbiotic dinoflagellate, *Symbiodinium* sp., and a brown alga, *Petalonia fascia*, respectively, were compared for inhibitory effects on delayed-type hypersensitivity in mice. The number of eosinophils at the site of inflammation and in peripheral blood was compared for the administration of peridinin and fucoxanthin applied by painting and intraperitoneally. Peridinin, but not the structurally-related fucoxanthin, significantly suppressed the number of eosinophils in both the ear lobe and peripheral blood. Furthermore, peridinin applied topically, but not administered intraperitoneally, suppressed the level of eotaxin in the ears of sensitized mice. Fucoxanthin weakly suppressed the concentration of eotaxin in ears only by intraperitoneal administration. Although both carotenoids inhibited the migration of eosinophils toward eotaxin, the inhibitory effect of peridinin was higher than that of fucoxanthin. Peridinin may be a potential agent for suppressing allergic inflammatory responses, such as atopic dermatitis, in which eosinophils play a major role in the increase of inflammation.

## 1. Introduction

Dinoflagellates are unicellular phytoplankton and are known to produce various bioactive secondary metabolites [1,2,3,4,5,6]. The genus, *Symbiodinium*, which belongs to the zooxanthellae, is a representative symbiont found in many marine invertebrates. They also produce unique and complex bioactive secondary metabolites [1,2,4] together with a peridinin. Peridinin is one of the carotenoids that is synthesized in dinoflagellates.

Carotenoids have numerous bioactivities. In particular, the marine carotenoid, fucoxanthin, has multiple functions and has been reported to have considerable potential for applications for improving human health [7,8,9,10,11,12]. For example, fucoxanthin induces the uncoupling of protein 1 expression in white adipose tissue mitochondria [7]. It also improves insulin resistance and decreases blood glucose levels [7]. Kim *et al.* [9] reported that fucoxanthin reduces the levels of pro-inflammatory mediators, including NO, PGE_2_, IL-1β, TNF-α and IL-6, via the inhibition of NF-κB activation and the suppression of MAPK phosphorylation in leukemic monocyte RAW264 cells. Sakai *et al.* [8] reported that fucoxanthin, astaxanthin, zeaxanthin and β-carotene significantly inhibit the antigen-induced release of β-hexosaminidase in basophilic leukemia 2H3 and mast cells and that these carotenoids also inhibit antigen-induced aggregation of the high affinity IgE receptor on mast cells. Further, Sakai *et al.* [10] reported that these carotenoids inhibit dinitrofluorobenzene-induced contact hypersensitivity in the ears by reducing the TNF-α and histamine levels in the ear.

**Figure 1:**
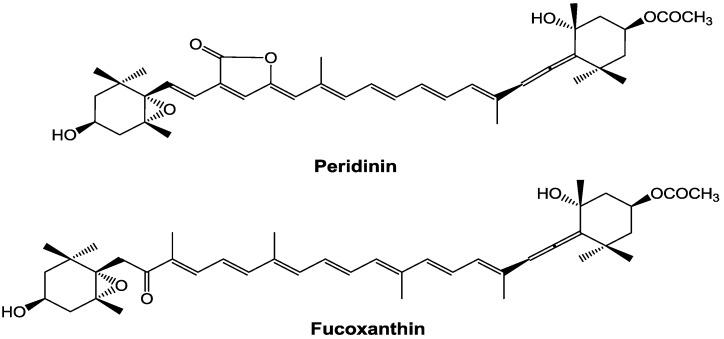
Chemical structures of peridinin and fucoxanthin.

In contrast, the bioactivities of peridinin, which has a structure similar to that of fucoxanthin (Figure 1), have not been well studied. Tsushima *et al.* [13] reported that peridinin has a strong inhibitory effect on Epstein–Barr virus early antigen activation in Raji cells at a lower concentration, but shows cytotoxicity to Raji cells at a higher concentration. Sugawara *et al.* [14] reported that peridinin induced apoptosis of human colorectal cancer cells by activating both caspase-8 and caspase-9.

Here, we examine the bioactivity of peridinin as a functional material for human health. Recently, the number of allergy sufferers has increased worldwide. In this paper, we examined the inhibitory effect of peridinin on delayed-type hypersensitivity in mice and compared it with that of fucoxanthin.

## 2. Results and Discussion

### 2.1. Effect of Peridinin on DTH in BALB/cAJc1 Mice

Eosinophils are well-known granulocytes that increase at the sites of inflammation as part of the delayed-type hypersensitivity (DTH) response. Eosinophils are reported to increase in response to picryl chloride (PCl; 2,4,6-trinitrochlorobenzene) in BALB/cAJc1 mice [15]. In that study, treatment with cyclophosphamide 2 days before sensitization resulted in marked blood and tissue eosinophilia. We applied the same experimental protocol to elicit the DTH response.

**Figure 2:**
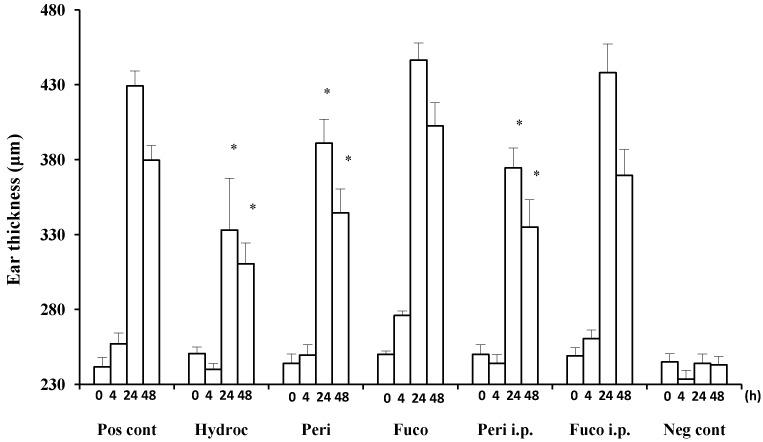
Suppression of delayed-type hypersensitivity (DTH) responses by peridinin and fucoxanthin in BALB/cAJc1 mice. Mice were sensitized with picryl chloride (PCl) after pretreatment with cyclophosphamide. Two weeks after 10 μg of peridinin or fucoxanthin or hydrocortisone in 10 μL of olive oil was painted onto the ears (per earlobe), 50 μg of peridinin or fucoxanthin in 100 μL of olive oil was administered i.p. (intraperitoneally) to these mice 3 h before the antigen challenge. Ear thickness was measured with a dial thickness gauge before (0 h) and after the antigen challenge (4, 24 and 48 h). Each value for each treatment group is expressed as the mean ± SD (*n* = 10). Pos cont, positive control (sensitized and challenged); Hydroc, hydrocortisone; Peri, peridinin; Fuco, fucoxanthin; Peri i.p., peridinin administered intraperitoneally; Fuco i.p., fucoxanthin administered intraperitoneally; Neg cont, negative control (sensitized, but not challenged). * An asterisk indicates a significant difference between the treatment group and the positive control (*p* < 0.01, Tukey–Kramer’s *post hoc* test). Asterisks are only shown for groups showing suppression compared to the positive control at 24 or 48 h after the antigen challenge.

Hydrocortisone strongly suppressed the DTH response at 24 and 48 h (22.4% and 18.2% suppression, respectively; Figure 2) after the antigen challenge. Peridinin also suppressed the DTH response in BALB/cAJc1 mice, both at 24 or 48 h after the antigen challenge by both routes of administration, painted onto ears (paint) (8.9% and 9.2% suppression, respectively; Figure 2) or administered intraperitoneally (i.p.) (12.7% and 11.7% suppression, respectively; Figure 2). In contrast, fucoxanthin did not suppress the DTH response at either 24 or 48 h after the antigen challenge (Figure 2).

Increased ear thickness is caused by the accumulation of lymphocytes, macrophages, neutrophils and eosinophils. Furthermore, macrophages stimulate the proliferation of fibroblasts. The accumulation of these cells to the site of antigen challenge results in the increase of ear thickness. It is suggested that peridinin reduced at least one of these factors. We focused on eosinophils, because they are typical white blood cells that increase in allergic reactions.

### 2.2. Induction of Tissue Eosinophilia in Peridinin-Treated Mice Sensitized with PCl

**Figure 3:**
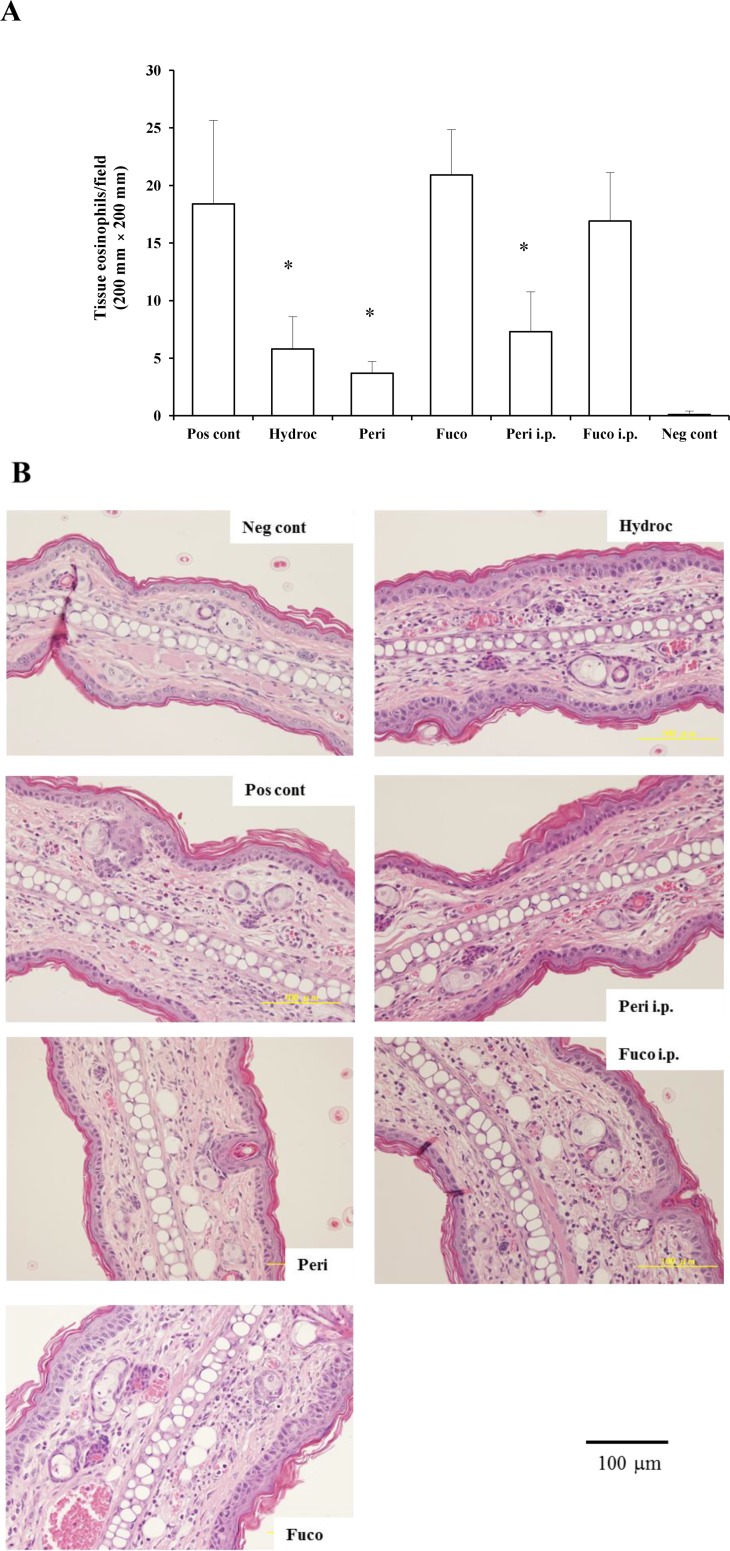
The effects of peridinin or fucoxanthin on the number of eosinophils in ear sections. Mice were sensitized with PCl, as described in the Experimental Section. Two weeks after the sensitization, 10 µg of peridinin, fucoxanthin or hydrocortisone was painted onto the ears and 50 µg of peridinin or fucoxanthin was administered i.p. to these mice 3 h before the antigen challenge. At 48 h after the antigen challenge, tissue specimens were fixed and stained with hematoxylin/eosin solution to count the number of eosinophils. (**A**) The number of eosinophils in each section from each group is shown. (**B**) Representative section photomicrographs from each group of mice are shown. Each value is expressed as the mean ± SD (*n* = 10). Pos cont, positive control; Hydroc, hydrocortisone; Peri, peridinin; Fuco, fucoxanthin; Peri i.p., peridinin intraperitoneally; Fuco i.p., fucoxanthin intraperitoneally; Neg cont, negative control. * An asterisk indicates a significant difference between the treatment group and the positive control (*p* < 0.01, Tukey–Kramer’s *post hoc* test).

We examined whether the migration of eosinophils to the site of inflammation was suppressed by administering peridinin or fucoxanthin. We measured the number of eosinophils in the ear section of BALB/cAJc1 mice and compared it to the number in the negative control group (sensitized, but not challenged with PCl) and the hydrocortisone group (68.5% suppression). Peridinin by either route of administration, paint (79.9% suppression) or i.p. (60.3% suppression), significantly decreased the number of eosinophils at the site of inflammation (ear section) at 48 h after antigen challenge. In contrast, fucoxanthin did not inhibit the number of eosinophils that migrated to the site of inflammation at 48 h after the antigen challenge (Figure 3A,B). Eosinophils were not observed in the negative control group without the challenge with PCl. Eosinophils were more abundant after the challenge of sensitized mice with PCl. In the group treated with hydrocortisone by painting at 3 h before the challenge, the number of eosinophils was lower than that of the positive control. Groups treated with peridinin either by painting or i.p. showed lower number of eosinophils than the positive control. There were no significant difference between the hydrocortisone-treated group and peridinin-treated group. In contrast, the fucoxanthin-treated group, either by painting or i.p., did not have a lower number of eosinophils compared with the positive control group.

### 2.3. Suppression of the Percentage of Eosinophils in Peripheral Blood in Peridinin-Treated Mice Sensitized with PCl

**Figure 4:**
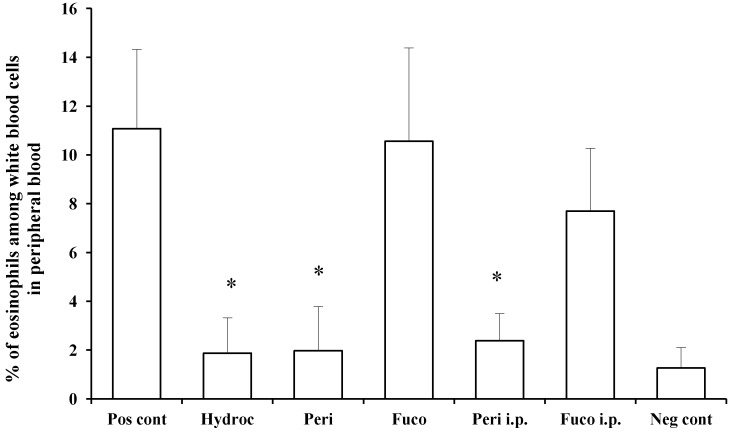
Effects of peridinin or fucoxanthin on the number of eosinophils in peripheral blood. Mice were sensitized with PCl, as described in the Experimental Section. Two weeks after the sensitization, 10 μg of peridinin, fucoxanthin or hydrocortisone was painted onto the ears and 50 μg of peridinin or fucoxanthin was administered i.p. to these mice 3 h before the antigen challenge. At 48 h after the antigen challenge, mice were bled from the retro-orbital plexus. The percentage of eosinophils among white blood cells was determined by making a smear on a slide glass for each mouse. Slide glasses were stained with Giemsa solution, and the percentage of eosinophils among white blood cells was estimated by counting at least 200 white blood cells from each sample. There was no significant difference in the total numbers of leukocytes among groups. Each value is expressed as the mean ± SD (*n* = 10). Pos cont, positive control; Hydroc, hydrocortisone; Peri, peridinin; Fuco, fucoxanthin; Peri i.p., peridinin intraperitoneally; Fuco i.p., fucoxanthin intraperitoneally; Neg cont, negative control. * An asterisk indicates a significant difference between the treatment group and the positive control (*p* < 0.01, Tukey–Kramer’s *post hoc* test).

We measured and compared the percentage of eosinophils among white blood cells in the peripheral blood of individual mice between the non-challenged and hydrocortisone-treated groups and found the effect of treatment to result in 83.1% suppression. Peridinin administered by either route, paint (82.2% suppression) or i.p. (78.4% suppression), significantly suppressed the number of eosinophils in peripheral blood at 48 h after the antigen challenge. In contrast, fucoxanthin did not suppress the number of eosinophils in peripheral blood at 48 h after the antigen challenge (Figure 4). These results suggest that peridinin, but not fucoxanthin, suppressed the proliferation of eosinophils. In this DTH model, serum levels of IFN-γ and IL-5 were increased 48 h after the antigen challenges, and peridinin did not suppress the serum level of either cytokine significantly in either route of administration (IFN-γ, negative control, 37.8 ± 14; positive control, 104.4 ± 28; peridinin painted, 94 ± 32; peridinin i.p., 72.6 ± 31. IL-5: negative control, 12.3 ± 8.5; positive control, 18.6 ± 3.5; peridinin painted, 25.0 ± 0.1; peridinin i.p., 23.2 ± 12. Results are expressed as the mean (picogram per millimeter) ± SD). The serum level of IL-17 was not increased in this experiment [16]. Thus, we hypothesized that the production of eotaxin, the most potent chemo-attractant of the eosinophils, may be suppressed at the site of inflammation.

### 2.4. Effects of Peridinin on Eotaxin Production

**Figure 5:**
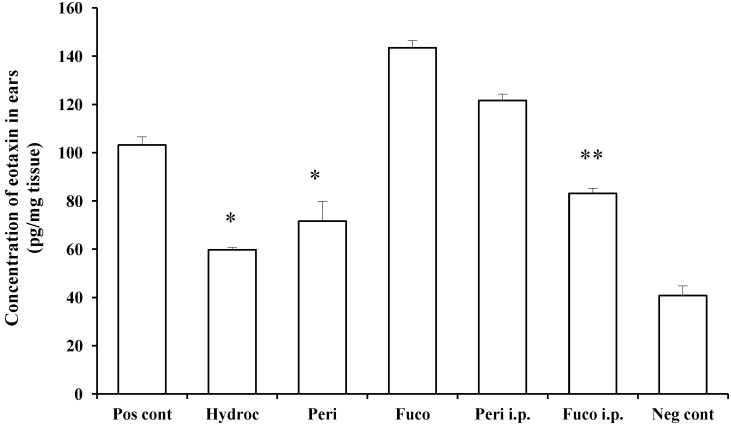
Peridinin inhibits eotaxin production in challenged skin. Mice were sensitized as described in the Experimental Section. At 48 h after the antigen challenge, ear lobes were removed, and the concentration of eotaxin in each ear lobe was measured as described in the Experimental Section. Each value is expressed as the mean ± SD (*n* = 3). Pos cont, positive control; Hydroc, hydrocortisone; Peri, peridinin; Fuco, fucoxanthin; Peri i.p., peridinin intraperitoneally; Fuco i.p., fucoxanthin intraperitoneally; Neg cont, negative control. * An asterisk indicates a significant difference between the treatment group and the positive control (*p* < 0.01, Tukey–Kramer’s *post hoc* test). ** There is a significant difference between Fuco i.p. and the positive control (*p* < 0.05, Tukey–Kramer’s *post hoc* test). Asterisks are shown only for treatment groups showing suppression.

Figure 5 shows that eotaxin production was stimulated by the challenge with PCl, and this was inhibited by peridinin following painting onto the ears (30.6% inhibition), but not by i.p administration. This inhibitory effect was comparable to that of hydrocortisone (42.1% inhibition). On the other hand, fucoxanthin weakly inhibited the production of eotaxin in mice administered i.p (19.4% inhibition), whereas it had no effect in the case of painting. These results suggest that metabolites of peridinin and fucoxanthin administered i.p. may have different effects on eotaxin-producing cells, such as endothelial cells, epithelial cells and macrophages. It is also possible that there is a difference between these two compounds in terms of penetration through the skin. These differences between peridinin and fucoxanthin remain to be clarified.

### 2.5. Inhibitory Effects of Peridinin and Fucoxanthin on the Chemotaxis of Eosinophils toward Eotaxin

**Figure 6:**
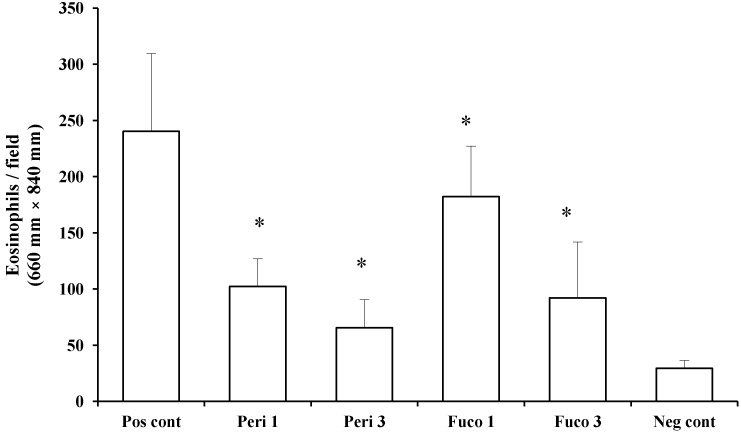
The effect of peridinin or fucoxanthin on the migration of eosinophils toward eotaxin in chemotaxis assays. Eosinophils prepared as described in the Experimental Section were suspended in Roswell Park Memorial Institute (RPMI) 1640 medium containing 0.1% bovine serum albumin (BSA) and placed in the Transwells of a 24-well chemotaxis chamber. The lower chamber contained RPMI 1640 medium containing 0.1% BSA and mouse eotaxin (final concentration: 20 ng/mL). Peridinin or fucoxanthin at a final concentration of 1 or 3 μg was added to each Transwell. Assay plates were incubated for 1 h at 37 °C in 5% CO_2_. After 1 h incubation, the cells that migrated across the filter to the lower chamber were counted. Each value is expressed as the mean ± SD (*n* = 18). Pos cont, positive control; Peri 1, 1 µg of peridinin; Peri 3, 3 μg of peridinin; Fuco 1, 1 μg of fucoxanthin; Fuco 3, 3 μg of fucoxanthin; Neg cont, negative control. * An asterisk indicates a significant difference between the treatment group and the positive control (*p* < 0.01, Tukey–Kramer’s *post hoc* test).

Next, we asked whether peridinin inhibits the migration of eosinophils using murine eosinophils isolated from IL-5 transgenic mice. Peridinin and fucoxanthin were tested in an eosinophil chemotaxis assay *in vitro*. Chemotaxis of eosinophils toward eotaxin (20 ng/mL) was suppressed by one and 3 µg/mL of peridinin (57.4% and 72.8% suppression, respectively; Figure 6) and by one and 3 µg/mL of fucoxanthin (24.2% and 61.7% suppression, respectively; Figure 6). Peridinin showed higher suppression of the migration of eosinophils toward eotaxin than did fucoxanthin. More than 95% of eosinophils were viable after one hour of incubation for the chemotaxis toward eotaxin. The viability of eosinophils was checked by trypan blue exclusion.

Although the painting of peridinin on earlobes decreased the level of eotaxin in the ears, the i.p. administration of peridinin did not have any effect on the production of eotaxin in the ears. In the peripheral blood, however, the number of eosinophils was suppressed significantly in either route of administration, suggesting that peridinin suppressed the number of eosinophils in the ears by reducing the number of eosinophils in the peripheral blood. Therefore, the level of eotaxin in the ears does not have a primary role in case of the i.p. administration of peridinin. Since peridinin did not change the serum level of IL-5 in either route of administration as described above, the decreased number of eosinophils in peripheral blood may be regulated by other factors.

In contrast, the number of eosinophils in peripheral blood was not influenced by fucoxanthin in either route of administration. Although fucoxanthin suppressed the migration of eosinophils *in vitro*, this effect seems to be marginal *in vivo*, because there is an increased level of eosinophils in the peripheral blood and a higher level of eotaxin is produced in the ears.

## 3. Experimental Section

### 3.1. Materials

Picryl chloride (PCl; 2,4,6-trinitrochlorobenzene) was purchased from Nacalai Tesque (Kyoto, Japan). Cyclophosphamide was purchased from Shionogi Pharmaceutical Co. (Osaka, Japan). Hydrocortisone was purchased from Sigma-Aldrich (St. Louis, MO, USA). Recombinant mouse eotaxin (CCL11) was purchased from ProSpec-Tany TechnoGene Ltd. (Ness Ziona, Israel). Peridinin and fucoxanthin were isolated from a dinoflagellate, *Symbiodinium* sp. (OTCL2A strain), and brown alga, *Petalonia fascia*, in culture, respectively.

### 3.2. Preparation of Peridinin

Peridinin was isolated from a cultured dinoflagellate, *Symbiodinium* sp. (OTCL2A strain). This *Symbiodinium* sp. was isolated from the giant clam, *Tridacna crocea*, by the author and cultured by a method reported previously [2]. The frozen cells (82.0 g from 132 L of culture) were homogenized in 70% EtOH (150 mL) with an Ultra-Turrax T25 homogenizer (Janke & Kunkel GmbH & Co. KG IKA-Labortechnik, Staufen, Germany), soaked for 3 days at 4 °C and centrifuged. The supernatant was collected; the precipitate was extracted twice with 70% EtOH (150 mL each). The ethanol extracts from the centrifugation and extractions were combined and evaporated *in vacuo*. The residue was suspended in water (100 mL) and extracted with EtOAc (3 × 200 mL). The combined EtOAc extracts were concentrated under reduced pressure and yielded an EtOAc-soluble fraction (903 mg). A portion (676 mg) of the EtOAc-soluble fraction was chromatographed on silica gel (60 mL of Silica Gel 60, Nacalai Tesque) and eluted with 120 mL of CH_2_Cl_2_:MeOH in fractions on a gradient of 99:1, 98:2, 96:4 and 92:8. A portion (199.2 mg) eluted from the last two extractions with CH_2_Cl_2_:MeOH (96:4 and 92:8; 264.8 mg) was then separated by chromatography on ODS (19 mL of Cosmosil 75C18-OPN, Nacalai Tesque) followed by eluting steps with 40 mL of 80% MeOH and 80 mL of 85% MeOH. The fraction eluted with 85% MeOH had a crude yield of 98.8 mg and, following separation by HPLC (COSMOSIL 5C18-AR-II, 20 mmφ × 250 mm, Nacalai Tesque) with 85% MeCN at a flow rate of 6.0 mL min^−1^, yielded peridinin (24.7 mg).

### 3.3. Preparation of Fucoxanthin

Fucoxanthin was extracted from the brown alga, *Petalonia fascia*, cultured as described [17]. A sample (54.5 g) of frozen *Petalonia fascia* was extracted four times with 500 mL of MeOH. The combined methanol extracts were evaporated *in vacuo*. The residue was dissolved in 100 mL of 90% MeOH and then partitioned with hexane (2 × 100 mL). The 90% MeOH layer was removed to a different vessel and evaporated. The residue was resuspended in 100 mL of H_2_O and then extracted with EtOAc (2 × 100 mL). The EtOAc-soluble fraction (602.1 mg) was applied to a silica gel column (12 mL of Silica Gel 60, Nacalai Tesque), and the column was eluted with 170 mL of 1:1 hexane:EtOAc to give seven fractions. The fifth fraction (32.4 mg) was then separated by chromatography on ODS (10 mL of Cosmosil 75C18-OPN, Nacalai Tesque), and elution was performed with 40 mL of 85% MeOH and 30 mL of 90% MeOH. Fucoxanthin with a yield of 12.8 mg was eluted in the fraction of 90% MeOH.

### 3.4. Animals

BALB/cAJc1 mice were purchased from CLEA Japan, Inc. (Osaka, Japan). IL-5 transgenic mice (C3H/HeN-TgN(IL-5)-Imeg) were developed by our group [18] and were maintained in our animal facility under specific pathogen-free conditions. Mice used in the experiments were all female, and BALB/cAJc1 mice were 8 to 10 weeks of age and IL-5 mice were 8 to 15 weeks of age at the time of the experiments. All experiments were performed under the ethical guidelines of Kochi University.

### 3.5. Sensitization and Challenge

Two days before sensitization with PCl, cyclophosphamide was injected subcutaneously (150 mg/kg in distilled water) to remove proliferating immunosuppressive cells [15]. After removing coat hair, the mice were immunized by painting their abdominal skin with 0.05 mL of 7% PCl in ethanol:acetone (3:1). Two weeks after sensitization, 10 μg of peridinin, or fucoxanthin or hydrocortisone was painted onto the ears. 50 μg of peridinin or fucoxanthin was administered i.p. to these mice 3 h before the antigen challenge, and 0.02 mL of 1% PCl in acetone:olive oil (1:4) was painted on each ear lobe to challenge the mice. Ear thickness was measured with a dial thickness gauge (Peacock G-1M, Ozaki Mfg. Co. Ltd., Tokyo, Japan) before and after the challenge and was expressed as the mean thickness of each ear in micrometers.

### 3.6. Tissue Eosinophil Counts

At 48 h after being challenged, mice were sacrificed, and the ears were removed and fixed with 4% paraformaldehyde in a 0.1 M Phosphate buffer, pH 7.2. Then, the ears were embedded in paraffin, and sections of ears were stained with hematoxylin/eosin solution. The number of eosinophils infiltrated into the dermis was examined at a magnification of 400×. At least 10 fields were examined for each ear lobe. The number of eosinophils was counted and expressed as the number of cells in a field (200 μm × 200 μm).

### 3.7. Percentage of Eosinophils in Peripheral Blood

At 48 h after the antigen challenge, mice were bled from the retro-orbital plexus. The percentage of eosinophils among the white blood cells was determined by making a smear on a slide glass for each mouse. Slide glasses were stained with Giemsa solution, and the percentage of eosinophils among the white blood cells was estimated by counting eosinophils and all white blood cells in the field until at least 200 white blood cells were counted in each sample. At a minimum, 10 slides were counted and the mean of the counts were taken for each group.

### 3.8. Cytokine Measurements by ELISA

Capture antibodies (50 μL of 1 mg/mL in 0.05 M sodium carbonate, pH 9.6) were added to coat each well of Immuno 96-microwell plates (9018, Corning Costar, Ithaca, NY, USA) and incubated overnight at 4 °C. After washing wells twice with 0.15 M sodium phosphate buffer, phosphate buffered saline (PBS), pH 7.2, containing 0.05% Tween 20 (PBS-T), blocking buffer (PBS containing 0.05% Tween 20 and 0.5% bovine serum albumin (BSA)) was added and incubated for 30 min at room temperature. After removing blocking buffer, diluted sera (5 times with blocking buffer) were added and incubated for 2 h at room temperature or at 37 °C. After washing wells with PBS-T three times, 50 μL of 0.5 μg/mL biotin-coupled antibodies (detection antibodies) against each cytokine were added to each well and incubated at room temperature for 45 min. After washing with PBS-T three times, streptavidin-horseradish peroxidase (Invitrogen, Camarillo, CA, USA) was added and incubated at room temperature for 45 min, according to the manufacturer’s protocol. After washing wells with PBS-T three times, the substrate solution containing freshly prepared 0.7 mg/mL of *o*-phenylenediamine dihydrochloride (WAKO Pure Chemical Industries Ltd., Osaka, Japan) in citric acid buffer, pH 5.0, containing 0.006% hydrogen peroxide was added and incubated for 10 min. Reactions were stopped by adding 50 μL of 10% sulfuric acid to each well. Cytokine concentration was estimated by measuring the optical density (OD) at 490 nm using a micro plate reader (Model 680, Bio-Rad, Hercules, CA, USA) and a standard curve. Each cytokine level is expressed as the mean (pg/mL) ± SD.

### 3.9. Antibodies Used in ELISA

Capture antibodies included: rat anti-mouse IFN-γ antibodies (1 mg/mL), clone R46A2 (Becton Dickinson, Mountain View, CA, USA); rat anti-IL-5 antibodies (1 mg/mL), clone TRFK5 (prepared by Shiro Ono, Osaka Ohtani University, Tondabayashi, Japan); and rat anti-IL17 antibodies (2 mg/mL), clone TC11-18H10.1 (Becton Dickinson). Detection antibodies included: rat anti-mouse IFN-γ antibodies, clone XMG1.2 (Becton Dickinson); rat anti-IL-5 antibodies, clone TRFK4 (Becton Dickinson); and rat anti-IL17 antibodies, clone TC11-8H4.1 (Becton Dickinson).

### 3.10. Measurement of Eotaxin in Skin

At 48 h after being challenged, mice were sacrificed, and the ears were removed. The ear from each mouse in each experimental group was homogenized with PBS containing 0.1% Tween 20 (100 μL/10 mg tissue), using a mixer mill (MM 300, Retsch, Haan, Germany) with zirconia beads (5 mm) for 2 min at 30 Hz. The homogenates were centrifuged at 12,000× *g* for 10 min. The supernatants were kept at −80 °C until being assayed. The concentration of eotaxin in the supernatants was measured by a Mouse Eotaxin ELISA kit (Ray Biotech, Inc., Norcross, GA, USA). The sensitivity of the eotaxin assay was >0.01 pg/mg tissue.

### 3.11. Eosinophil Preparation

Enriched preparations of eosinophils were obtained from the peripheral blood of IL-5-transgenic mice. Eosinophil-enriched cells were obtained by the Percoll density gradient separation method described previously [19], with modification. Briefly, isotonic Percoll was prepared using a 10× solution of Krebs Ringer PBS (KRP; 10 mM sodium phosphate buffer, pH 7.5, containing 154 mM NaCl, 6 mM KCl and 1 mM MgCl_2_) and diluted with KRP to achieve concentrations of 60%, 70% and 80%. In 15-mL conical polypropylene tubes (BD Falcon 352096, Becton, Dickinson and Company, Franklin Lakes, NJ, USA), 2 mL of cell suspensions of 20 to 50 × 10^6^ cells in KRP were placed, followed by the careful layering of aliquots (2.5 mL) of each concentration of Percoll solution starting with the lowest concentration at the bottom. The tubes were spun at 1000× *g* for 20 min at room temperature. Eosinophils were extracted from the 70% to 80% Percoll fractions by removing B and T lymphocytes using anti-B220- and anti-Thy1.2-coupled Dynabeads (DYNAL A.S., Oslo, Norway). Briefly, lymphocytes bound to these beads were removed using a permanent magnet. Unbound cells were used as an eosinophil-enriched fraction. To identify eosinophils, aliquots were removed and assessed using eosinophil peroxidase staining as described previously [19]. More than 93% of the cells prepared by this method were eosinophils.

### 3.12. Chemotaxis Assay toward Eotaxin

Eosinophils (1.3 × 10^6^ cells/mL) were suspended in RPMI 1640 medium containing 0.1% BSA and were placed in the upper well (Transwell) of a 24-well chemotaxis chamber (KURABO Co., Osaka, Japan). Transwells with 5 μm pores were inserted into each well, and 4 × 10^5^ cells in 300 μL of RPMI 1640 medium containing 0.1% BSA were added to the upper chamber. The lower chamber was set with 800 μL of RPMI 1640 medium containing 0.1% BSA and mouse eotaxin (20 ng/mL). Then, 3 or 1 μg of peridinin or fucoxanthin (*n* = 6) was added to each Transwell. Assay plates were incubated for 1 h at 37 °C in 5% CO_2_. Cells that migrated across the filter to the lower chamber were counted, and the results were expressed as the number of cells in a field of 660 μm × 840 μm. For each group, eosinophils in three fields for each well were counted, and results were reported as the mean of 18 fields (cell number ± SD).

### 3.13. Statistical Analysis

For all experiments, ANOVA was performed, and the Tukey–Kramer *post hoc* test was used to identify significantly different means. A *p*-value <0.01 was considered statistically significant.

## 4. Conclusions

In conclusion, peridinin suppressed DTH responses in mice. Peridinin also suppressed the numbers of eosinophils in ear tissues and peripheral blood. When painted on the ears, peridinin inhibited both the migration of eosinophils toward eotaxin and the production of eotaxin in ears. However, the suppressive effect of peridinin on the production of eotaxin was not observed when administered i.p*.* A structurally related carotenoid, fucoxanthin, inhibited the migration of eosinophils toward eotaxin only *in vitro* and did not suppress the DTH response. The major structural difference between peridinin and fucoxanthin is the presence of a butenolide moiety in peridinin. The butenolide moiety of peridinin may be important for suppressing these effects on eosinophils and for the production of eotaxin. Comparison of the inhibitory effects of peridinin and other carotenoids with the butenolide moiety remains to be clarified.

As described above, peridinin may ameliorate the allergic responses in which eosinophils play a major role in inflammation responses, such as asthma or atopic dermatitis.

## References

[1] Nakamura H., Asari T., Murai A., Kan Y., Kondo T., Yoshida K., Ohizumi Y. (1995). Zooxanthellatoxin-A, a potent vasoconstrictive 62-membered lactone from a symbiotic dinoflagellate. J. Am. Chem. Soc..

[2] Onodera K., Nakamura H., Oba Y., Ohizumi Y., Ojika M. (2005). Zooxanthellamide Cs: Vasoconstrictive polyhydroxylated macrolides with the largest lactone ring size from a marine dinoflagellate of *Symbiodinium* sp.. J. Am. Chem. Soc..

[3] Yasumoto T. (2005). Chemistry, etiology, and food chain dynamics of marine toxins. Proc. Jpn. Acad. Ser. B.

[4] Kita M., Ohno O., Han C., Uemura D. (2010). Bioactive secondary metabolites from symbiotic marine dinoflagellates: Symbiodinolide and durinskiols. Chem. Rec..

[5] Ianora A., Bentley M.G., Caldwell G.S., Casotti R., Cembella A.D., Engström-Öst J., Halsband C., Sonnenschein E., Legrand C., Llewellyn C.A. (2011). The relevance of marine chemical ecology to plankton and ecosystem function: An emerging field. Mar. Drugs.

[6] Ciminiello P., Dell’Aversano C., Dello Iacovo E., Fattorusso E., Forino M., Grauso L., Tartaglione L., Guerrini F., Pezzolesi L., Pistocchi R. (2012). Isolation and structure elucidation of Ovatoxin-a, the major toxin produced by *Ostreopsis ovate*. J. Am. Chem. Soc..

[7] Miyashita K. (2009). The carotenoid fucoxanthin from brown seaweed affects obesity. Lipid Technol..

[8] Sakai S., Sugawara T., Matsubara K., Hirata T. (2009). Inhibitory effect of carotenoids on the degranulation of mast cells via suppression of antigen-induced aggregation of high affinity IgE receptors. J. Biol. Chem..

[9] Kim K.N., Heo S.J., Yoon W.J., Kang S.M., Ahn G., Yi T.H., Jeon Y.J. (2010). Fucoxanthin inhibits the inflammatory response by suppressing the activation of NF-κB and MAPKs in lipopolysaccharide-induced RAW 264.7 macrophages. Eur. J. Pharmacol..

[10] Sakai S., Sugawara T., Hirata T. (2011). Inhibitory effect of dietary carotenoids on dinitrofluorobenzene-induced contact hypersensitivity in mice. Biosci. Biotechnol. Biochem..

[11] Peng J., Yuan J.P., Wu C.F., Wang J.H. (2011). Fucoxanthin, a marine carotenoid present in brown seaweeds and diatoms: Metabolism and bioactivities relevant to human health. Mar. Drugs.

[12] D’Orazio N., Gemello E., Gammone M.A., de Girolamo M., Ficoneri C., Riccioni G. (2012). Fucoxantin: A treasure from the sea. Mar. Drugs.

[13] Tsushima M., Maoka T., Katsuyama M., Kozuka M., Matsuno T., Tokuda H., Nishino H., Iwashima A. (1995). Inhibitory effect of natural carotenoids on Epstein-Barr virus activation activity of a tumor promoter in Raji cells. A screening study for anti-tumor promoters. Biol. Pharm. Bull..

[14] Sugawara T., Yamashita K., Sakai S., Asai A., Nagao A., Shiraishi T., Imai I., Hirata T. (2007). Induction of apoptosis in DLD-1 human colon cancer cells by peridinin isolated from the dinoflagellate, *Heterocapsa triquetra*. Biosci. Biotechnol. Biochem..

[15] Satoh T., Chen Q.-J., Sasaki G., Yokozeki H., Katayama I., Nishioka K. (1997). Cyclophosphamide-induced eosinophilia in contact sensitivity: Mechanism of hapten-induced eosinophil recruitment into the skin. Eur. J. Immunol..

[16] Ono S., Okayama H. (2013).

[17] Kimiya T., Ohtani K., Satoh S., Abe Y., Ogita Y., Kawakita H., Hamada H., Konishi Y., Kubota S., Tominaga A. (2008). Inhibitory effects of edible marine algae extracts on degranulation of RBL-2H3 cells and mouse eosinophils. Fish. Sci..

[18] Tominaga A., Takaki S., Koyama N., Katoh S., Matsumoto R., Migita M., Hitoshi Y., Hosoya Y., Yamauchi S., Kani Y. (1991). Transgenic mice expressing a B cell growth and differentiation factor gene (interleukin5) develop eosinophilia and autoantibody production. J. Exp. Med..

[19] Watanabe Y., Hashizume M., Kataoka S., Hamaguchi E., Morimoto N., Tsuru S., Katoh S., Miyake K., Matsushima K., Tominaga M. (2001). Differentiation of eosinophils characterized by hyaluronic acid binding via CD44 and responsiveness to stimuli. DNA Cell Biol..

